# Goods, causes and intentions: problems with applying the doctrine of double effect to palliative sedation

**DOI:** 10.1186/s12910-021-00709-0

**Published:** 2021-10-19

**Authors:** Hannah Faris, Brian Dewar, Claire Dyason, David G. Dick, Ainsley Matthewson, Susan Lamb, Michel C. F. Shamy

**Affiliations:** 1 Ottawa Hospital Research Institute, Ottawa, Canada; 2 Department of Medicine, University of Ottawa, Ottawa, Canada; 3 Department of Philosophy, University of Calgary, Calgary, Canada; 4 Canadian Centre for Advanced Leadership, Haskayne School of Business, University of Calgary, Calgary, Canada; 5 Department of Innovation in Medical Education, University of Ottawa, Ottawa, Canada; 6 The Ottawa Hospital, Ottawa, Canada

**Keywords:** Doctrine of double effect, Palliative sedation, Ethics, End-of-life care

## Abstract

**Background:**

Palliative sedation and analgesia are employed in patients with refractory and intractable symptoms at the end of life to reduce their suffering by lowering their level of consciousness. The doctrine of double effect, a philosophical principle that justifies doing a “good action” with a potentially “bad effect,” is frequently employed to provide an ethical justification for this practice.

**Main text:**

We argue that palliative sedation and analgesia do not fulfill the conditions required to apply the doctrine of double effect, and therefore its use in this domain is inappropriate. Furthermore, we argue that the frequent application of the doctrine of double effect to palliative sedation and analgesia reflects physicians’ discomfort with the complex moral, intentional, and causal aspects of end-of-life care.

**Conclusions:**

We are concerned that this misapplication of the doctrine of double effect can consequently impair physicians’ ethical reasoning and relationships with patients at the end of life.

## Background

Palliative sedation is an accepted but rarely used practice in palliative care in which medications are used in a monitored fashion to achieve a state of decreased or absent awareness when that is felt to be the only way of relieving a patient’s otherwise intractable suffering [1]. Palliative analgesia produces a similar result using analgesic rather than sedative medications [2]. Palliative sedation and analgesia are frequently criticized by those who argue that it hastens or even causes death, though many experts in palliative care disagree with this characterization [3, 4]. It is interesting then, that a classic principle of Catholic ethics known as the doctrine of double effect is frequently applied to justify this practice [5]. Classically, the doctrine of double effect is used to justify an action that produces a good effect – in this case the relief of suffering – even if it may produce a bad effect, for example hastening death. The discussion around whether and how the doctrine can be applied to palliative sedation is ongoing in the literature [6–9].

In this paper, we argue that the doctrine of double effect is inappropriately and harmfully applied because the conditions of the doctrine of double effect are not met in cases of palliative sedation. We are not for or against the doctrine itself, but are interested in its application in this case. We also show that the doctrine of double effect is referenced much more commonly and specifically than would be expected in relation to palliative sedation in the medical literature. Finally, we explore why the doctrine of double effect is so strongly linked to palliative sedation, and propose that this reflects the medical community’s discomfort with the (moral, medical, political) complexities of end-of-life decision-making. These complexities are increasingly important given the prominence of end-of-life care and assisted dying in the public consciousness [10, 11].

## The doctrine of double effect in medicine

The doctrine of double effect is an ethical device derived from St. Thomas Aquinas’ justification of killing in self-defense. Aquinas argued that an act of self-defense is justifiable even if the attacker is killed when the intention of the act is to save one’s self, and not to kill the attacker [12]. For Aquinas, it is the intention of the action that determines whether it is ethical. An action is permissible if it is intended to produce a “good” effect even though it might produce an unintended “bad” effect. Subsequently, ethicists have further elaborated a set of conditions that describe when the use of the doctrine of double effect applies, and hence justifies an action. These are:

1. All reasonable and less risky alternatives have been exhausted.
2. There is one action with at least two foreseeable effects.
3. The act itself is good (or at least neutral).
4. One effect is bad while the other is good.
5. The good effect is the discrete event towards which one is aiming (i.e., one’s intention-in-acting or the end of the act), not one’s further intention (i.e., the end of the agent).
6. These good and bad effects are not mediated by intervening agents, but flow immediately from the act.
7. One foresees the bad effect but intends only the good effect.
8. The bad effect is not the means by which the good effect is accomplished.
9. The act is proportionate in these two senses:
  The means employed are proportionate to the end (means-end proportionality)
  The potential benefits are proportionate to the potential harms (end-end proportionality) [6].

According to these criteria, the doctrine of double effect could be used to justify everyday medical practices, such as prescribing an antibiotic to treat a urinary tract infection, even if it produces an allergic reaction. The action is itself a good one, the bad consequence (the allergic reaction) is not the cause of the good consequence (treating the infection), the bad consequence is recognized at the time of prescribing, and the goal of eliminating the infection is felt to outweigh the potential risk of an allergic reaction. Indeed, this logic applies to many (if not all) decisions made in everyday medical practice. However, it seems that the doctrine of double effect is almost never used to justify everyday practices and is overwhelmingly used to justify palliative sedation and other end of life care.

To confirm this hypothesis, we performed a bibliometric analysis seeking to identify all references to the doctrine of double effect in the medical literature. In accordance with standard practices of bibliometrics [13]. We searched the PubMed database to identify all mentions of the double effect from its initiation up to 17 July 2020. We did not perform a systematic review as our objective was not to analyze these articles but rather to simply count mentions. We identified 627 references to the doctrine of double effect, of which the vast majority (432 articles, 68.9%) were related to topics in end-of-life care, including euthanasia (260, 41.5%), palliative sedation/analgesia (104, 16.6%), and other palliative care (68, 10.8%) (Table 1). Moreover, ethical discussions surrounding palliative sedation nearly always referenced the doctrine of double effect [14–19].

**Table 1:** Articles citing the doctrine of double effect by topic

Period	Total results	Euthanasia	Palliative sedation/analgesia	Other palliative care/end of life	Abortion	Other reproductive health	Conjoined twin separation	Biomedical research	Dual use technology	Philosophical analysis of the doctrine	Organ donation	Other
1970–1979	40	9	0	1	28	1	1	0	0	0	0	0
1980–1989	46	20	2	7	10	2	1	0	0	2	0	2
1990–1999	313	178	41	33	20	5	5	11	0	7	6	7
2000–2009	158	41	41	18	9	7	22	4	0	5	5	6
2010–2020	70	12	20	9	2	6	1	1	0	0	0	4
Total Articles	627	260	104	68	69	21	30	16	15	14	11	19
Percentage (%)	100	41.5	16.6	10.8	11.0	3.5	4.8	2.6	2.4	2.2	1.8	3.0

Of the 629 articles identified in the search, 2 (both from 2010 to 2020) were excluded for not relating to the doctrine

The remaining 627 articles were grouped by topic and decade of publication

This strong association between the doctrine of double effect and end-of-life care is even more unexpected because, as we will argue, palliative sedation does not fulfill the ethical criteria required to invoke the doctrine of double effect. Palliative sedation does not fulfill these criteria because: it is not always clear which outcomes are good and bad at the end of life, it is not evident that an action taken at the end of life truly causes an outcome, and physicians may directly or indirectly intend to cause or hasten death in patients who are terminally ill and experiencing intractable suffering.

## Main text

### Why the doctrine does not apply: goods

For the doctrine to apply to any given moral scenario, an action must have two potential effects: one that is good, and one that is bad. The use of the doctrine in reference to palliative care would suggest that treating suffering is a good and that causing unconsciousness, and potentially death, is bad. Moreover, the good effect must be felt to outweigh the potential bad effect. However, determining whether a given consequence is good or bad depends upon some moral framework or approach that explicitly identifies *goods* and *bads*. It is not clear which commonly cited moral framework would recognize the relief of suffering as a good that would outweigh potentially causing or hastening death.

For example, Thomas Aquinas would not have considered the relief of suffering a good that would offset a risk of causing death. For Aquinas, there were only four so-called “intrinsic goods”: human life, human procreation, human knowledge, and human sociability [20]. The avoidance or relief of suffering, pain, or sadness do not appear on Aquinas’ list of intrinsic goods. The destruction of life produced by palliative analgesia or sedation would trump the compassionate treatment of that patient’s suffering because one is an intrinsic good and the other is not. Consequently, risking life for the sake of treating suffering would not qualify for double effect reasoning under Aquinas’ moral framework.

Similarly, historically important moral frameworks from prominent philosophers would not support the use of the doctrine for palliative sedation. Under the philosophy of Immanuel Kant, the relief of suffering would not have proportionately trumped the production of unconsciousness, let alone hastening death [14, 21]. The ninth criterion of proportionality requiring the balancing of benefits and harms would not be satisfied. From the utilitarian perspective of Jeremy Bentham, the two most important principles were to produce pleasure and relieve pain; therefore palliative sedation, with the intent of relieving suffering, would likely have been acceptable even if it did induce unconsciousness or even death [22]. Therefore, there would be no bad effect to balance the good effect of relieving suffering (fourth criterion), and the doctrine would not be required to justify palliative sedation. For the above reasons, both Kant and Bentham would not have been satisfied that palliative sedation qualifies for double effect reasoning.

Contemporary autonomy-based reasoning privileges patients’ preferences about health care choices. For example, in its Expert Panel on End-of-Life Decision-Making, the Royal Society of Canada declared that “What matters, on the background of the autonomy-based ethical rationale laid out in this Report, is whether the result of the occurring assistance, namely the death of the patient, is what the patient desires” [23]. By this logic, patients who welcome death may be treated with palliative sedation without concern for causing death, given that both the relief of suffering and death would be considered good outcomes by those patients. Recent research in the UK indicates public support for palliative sedation and analgesia as an option in end of life care [24]. Patients who do not wish to risk death would refuse palliative sedation, obviating the need for the doctrine of double effect. These data suggest that for many people, the relief of suffering would be a desirable end even at the cost of life.

Indeed, physicians and nurses who treat dying patients may not see death as a bad effect. In a qualitative study of Australian physicians’ opinions on palliative sedation, one doctor said “if your only concern is to relieve pain, the corollary is that death is irrelevant, so why would you be concerned about not hastening death? … Death is the most effective way you can be sure the patient isn’t receiving any pain” [25]. As Allmark et al. have argued, “Once a patient is diagnosed as dying, his death is no longer something the health care team seeks to avoid. This change in attitude to a patient’s death is central to end-of-life care. And it appears to undermine the relevance of the doctrine of double effect” [26].

None of the major moral systems frequently discussed in bioethics clearly delineates the treatment of suffering as a good that outweighs the death of the patient as an evil. Since the double effect can only speak to actions that are considered to have good and bad effects, the practices of palliative sedation do not represent an appropriate subject.

### Why the doctrine does not apply: causes

For a scenario to justify the doctrine of double effect, the action must cause both the good and bad effects, and the bad effect of an action must not be the cause of the good effect. However, determining causation for actions at the end of life is very difficult. Therefore, applying the doctrine to palliative sedation is problematic because of the weakness of the causal arguments and empiric evidence that tie the action to its effects.

The application of the doctrine to palliative sedation seems to rely on the assumption that palliative sedation hastens death. However, the available evidence suggests that analgesia and sedation do not hasten death [27]. A 2003 review did not find any association between opioid exposure or sedation and shortened life [28]. In a multicenter, observational, prospective, nonrandomized population-based study, an Italian group found that palliative sedation in patients with advanced cancer was not associated with shortened life [29]. A Japanese group found similar results in a multi-centre prospective cohort study [30]. Some studies have suggested that the practices of palliative care can actually prolong life [28, 31] including in terminally ill cancer patients [32].

Critics of this literature argue that it provides weak evidence in defense of palliative sedation due to methodological issues and that a prospectively randomized, double blinded trial has not been performed [9, 26, 33] The lack of such data further emphasizes that, given the state of available knowledge, it is very difficult to establish medically plausible causal relations at the end of life. As a palliative care physician reported in a qualitative study, “In a dying patient, it may be very difficult to foresee whether the administration of sedation will hasten death, or merely coincide with an ‘imminent’ death due to the underlying cause” [34] The medications frequently used in palliative care have potentially dangerous side effects, but the chance of spontaneous death at any moment is also very high [23] This scenario produces persistent uncertainty that is deeply troubling for many physicians, and which may drive them towards seeking solace in the doctrine of double effect, albeit inappropriately.

### Why the doctrine does not apply: intentions

For the doctrine of double effect to apply to an action, the actor must intend to cause the good effect and not cause the bad effect. In the case of palliative sedation, the death of the patient must not be intended by the physician. How intentions are framed and formulated can greatly impact whether actions such as palliative sedation can be seen to fit double effect reasoning [7] A recent approach has distinguished intention by scope: the intention of the immediate act (intention-in-acting) versus the intention towards further consequences of the act (further intention) [6] In this framework, the double effect applies to the intention-in-acting rather than the further intention, as stated in the fifth criterion. Here we will argue on the basis of physician interviews and surveys that intentions cannot always be so clearly delineated. Many physicians have multiple intentions that can include intending or partially intending the death of the patient when administering palliative sedation. Therefore, the doctrine would not apply due to the fifth and seventh criteria not being met.

In considering physicians’ intentions in end-of-life care, Dr. Timothy Quill has suggested that “We would do well to look beneath the idealized, sanitized intentions espoused by many medical ethicists to the actual experience of doctors and patients” [35]. Quill is alluding to the fact that physicians and nurses involving in the care of the very sick and dying may intend to bring about the death of their patients as a means of relieving their suffering. Indeed, empirical approaches to documenting physicians’ intentions would support this hypothesis. While this kind of analysis is dependent upon “what physicians say (1) they remember (2) they thought (3) they did (4)” [36], there is as yet no better way of exploring intentions. Existing moral and legal frameworks also create a strong bias against admitting that the death of a patient was an intended effect of any physician’s action. Therefore, any statement to that effect must be considered especially powerful.

Many different studies from around the world support the notion that physicians and nurses often intend, or at least do not *not* intend, to cause death in terminally ill, suffering patients. Survey studies from several countries suggest that physicians often intend to bring about their patients’ deaths through palliative analgesia and sedation. In one study of American physicians, 39% reported that they had sedated patients with the intention of hastening death [37]. An Australian study found that 36% of general surgeons admitted using drugs at doses greater than necessary to relieve symptoms, with the intention of hastening death [38]. A Dutch survey found that 17% of physicians had prescribed palliative sedation with the explicit intention of hastening death, while another 47% had prescribed palliative sedation with the “partial intention” of hastening death [39]. A Japanese survey found that 38% of palliative care physicians prescribed palliative sedation with the explicit intention to maintain unconsciousness until death, with 11% reporting they intended to shorten survival to some extent [40]. Interviews with Australian general practitioners and palliative care physicians support the notion that intentions surrounding end-of-life care are multifaceted. One palliative care physician identified the integration of multiple intentions into the action of palliative sedation: “while the base, the prime intent, is to deal with the agonal state of the patient, there is no doubt that it also deals with our distress, and the family distress, and I don’t think you can separate those things, they are an intimate part of it” [36].

Therefore, it is difficult to support the notion that all physicians, nurses, family members and patients view the arrival of death as a “bad” effect of palliative sedation, nor as an unintended consequence. In the absence of an appropriate structure of goods, or established causation, or simple intentions, the application of the doctrine to palliative analgesia and sedation appears to be inappropriate. Additionally, and in line with the argumentation above, it is important to note that euthanasia and physician-assisted suicide would not qualify for double effect reasoning either. In the case of physician assisted suicide, causing death is not necessarily considered a bad effect, is explicitly intended, and is a means of achieving the good effect, of relieving suffering. Therefore, we now must ask why the doctrine of double effect is so frequently and consistently referenced in relation to end of life care.

## Why the doctrine of double effect is applied to palliative care

Why is consideration of palliative sedation so frequently associated with the doctrine of double effect? It is not because the doctrine’s concepts specifically apply to palliative care: as we have argued, the doctrine’s requirements are unmet in relation to end-of-life care. Rather, physicians may appeal to the doctrine of double effect as a means of managing their complex intentions in end of life care and to address the “moral distress” and moral and legal consequences of these complexities [5]. An Australian palliative care physician described palliative sedation as “well-intentioned self-deception… the intention would be to hasten the person’s death… while satisfying oneself that one is not providing a lethal bolus” [36]. Many physicians believe that they should never intend to hasten the death of their patients; consequently they apply the doctrine to sanitize their own intentions and avoid addressing the subjective moral assessments that determine goods at the end of life.

Alternatively, physicians may also appeal to the doctrine of double effect to justify their intentions to others. As Billings explains, “the rule of double effect has the important practical function of sanctioning appropriate symptom relief, including palliative sedation, while minimizing fears of accusations of PAS [physician-assisted suicide] or VAE [voluntary active euthanasia]” [5]. The importance of differentiating their practices from euthanasia is particularly important for palliative care physicians. Palliative care physicians tend to be vocal opponents of euthanasia and physician-assisted suicide, likely because they do not want to be accused or even suspected of engaging in physician-assisted suicide. In fact, many members of the palliative care community argue that optimal palliative care would make physician-assisted suicide unnecessary [41].

And yet, frequent references to the doctrine of double effect in the context of palliative care reinforce the notion that it hastens death. Forbes and Huxtable relate this phenomenon to “poor knowledge and inappropriate attitudes about the use of opioids at the end of life, believing, for instance, that addiction and tolerance are inevitable, respiratory depression limits dose escalation and that opioids shorten life” [42]. There is little empiric evidence to support this belief, and palliative care physicians contend that it is extremely unlikely that a properly prescribed dose of morphine near the end of life should play any causative role in that patient’s death [19]. Ultimately, much of this anxiety may be related to the inherent uncertainty surrounding causation at the end of life. Thus, invoking the doctrine may serve to resolve an epistemic (knowledge-related) problem, rather than an ethical one: we can’t know what the causes of our actions will be, but regardless of what happens the doctrine of double effect would justify them, or so the thinking might go. However, this epistemic problem is predicated on the “underlying predisposition to view death as a logical and moral error,” which does not seem to be the case. However this conflation reflects the interrelatedness of notions of goods, causes and intentions in physicians’ applications of the doctrine of double effect [43].

## Consequences of invoking the doctrine in palliative care

What are the consequences of invoking the doctrine in palliative scenarios? First, the doctrine is a form of “ethical reductionism” that appeals to physicians’ algorithmic intuitions to simplify clinical decision making [14], which might produce a “blocking effect” on the consideration of the ethical complexities surrounding palliative care. Such oversimplification could lead to the impoverishment of the ethical reasoning, and clinical care, that physicians provide to all patients.

Second, the doctrine encourages physicians to conceive of themselves as rational beings following rules and guidelines, rather than as complex, imperfect, biased and sensitive people who necessarily bring their own values and expectations into every clinical encounter. The doctrine provides no room for physicians to consider that they may have multiple intentions for providing care in a particular way to a particular patient. As Quill suggests, “being more forthright and explicit about our intentions and responsibility in working with dying patients” may be the key to restoring medical care in an increasingly dehumanized, academicized and commercialized context [35].

Third, the application of the doctrine to end of life care encourages physicians to develop a sense of moral detachment from the consequences of their actions, and from their patients. The doctrine dissuades physicians from engaging with their patients as equally complex, imperfect, biased and sensitive people; rather, it encourages a separation by seeing patients as vessels for good and bad effects, some intended and some not. This effect is unjustifiable, especially at the end of life.

Fourth, restricting the use of the doctrine to end-of-life care neglects that everyday medical decisions carry with them the risk of adverse and even fatal effects. The casual prescribing of opioids for pain relief without consideration of the risk of addiction has had fatal results within the ongoing opioid crisis, with 2861 opioid related deaths in 2016 in Canada [44]. Similarly, polypharmacy is strongly associated with mortality [45], especially among the elderly. In both cases, what might seem like straightforward clinical decisions can lead to tragic results when the potential negative effects of initiating treatment are not considered. Deeper reflection on the intentions and reasons behind medical decisions should not be limited to end-of-life care.

## Conclusions

The doctrine of double effect is used to justify actions that have intended “good” effects and unintended “bad” effects. In medicine, it is predominantly applied to justify the use of analgesia and sedation at the end of life, when medical interventions are feared to potentially hasten death. We have argued that this application of the doctrine is inappropriate, in that palliative care does not meet the requirements necessary to invoke the doctrine. We have concluded that the frequent invocation of the doctrine in palliative scenarios reflects physicians’ discomfort with the complex moral, intentional, and causal properties of end-of-life care. Physicians need to look beyond the doctrine to consider their own values and intentions so as to provide the more honest and ethical care possible to their patients.

## Data Availability

The data analyzed during the bibliometric analysis is available from the corresponding author for reasonable requests.

## Abbreviation

CPST: Continuous Palliative Sedation Therapy
